# Non-coding RNA influences in dementia

**DOI:** 10.1016/j.ncrna.2018.09.002

**Published:** 2018-09-29

**Authors:** Duncan Ayers, Charles Scerri

**Affiliations:** a Centre for Molecular Medicine and Biobanking, University of Malta, Msida, MSD 2080, Malta; b Faculty of Biology, Medicine and Health Sciences, The University of Manchester, Manchester, M13 9PL, United Kingdom; c Dept. of Pathology, Faculty of Medicine and Surgery, University of Malta, Msida, MSD 2080, Malta

**Keywords:** Non-coding RNA, ncRNA, Dementia, miRNA, microRNA, lncRNA

## Abstract

Dementia is a complex clinical syndrome characterised by progressive decline in cognitive function. It usually presents itself as impairment in memory, loss of judgement, abstract thinking and other disturbances that are severe enough to interfere with activities of daily living. It has long been considered as one of the major challenges at present posing an ever-increasing demand on global health and social care systems. Of all the different forms of dementia, Alzheimer's disease (AD) is the most common. The term non-coding RNA (ncRNA) refers to RNA sequences which do not have the ability to be translated into proteins and therefore mainly fall within the realm of the recently acknowledged ‘dark matter’ of the genome. This genomic dark matter encompasses a whole spectrum of differing ncRNA families such as microRNAs (miRNAs), long non-coding RNAs (lncRNAs), PIWI-interacting RNAs (piRNAs), transfer RNAs (tRNAs), small nuclear RNAs (snoRNAs) and circular RNAs (circRNAs), to name but a few. Consequently, due to the widespread influences of miRNAs and lncRNAs across all disease pathways, it is of critical importance for researchers in the field of dementia to focus their attention on possible ncRNA-induced pathogeneses, with the ultimate goal of identifying novel diagnostic procedures and drug targets, together with the development of novel therapies to control such a devastating mental condition in the patient population.

## 1 Introduction

Dementia is a complex clinical syndrome characterised by progressive decline in cognitive function. It usually presents itself as impairment in memory, loss of judgement, abstract thinking and other disturbances that are severe enough to interfere with activities of daily living. It has long been considered as one of the major challenges at present posing an ever-increasing demand on global health and social care systems. It is estimated that the worldwide cost of dementia reached US$ 1 trillion in 2018; accounting to more than one per cent of the world's gross domestic product [1]. A significant percentage of these costs occur in North America and Western Europe and are attributed to informal care, community and residential care and the direct expenditure in primary and secondary medical care [2].

The World Health Organization identified dementia as being responsible for more years lived with disability in people older than 60 years (11.2%) compared to stroke (9.5%), cardiovascular disease (5%) or all forms of cancer (2.4%) [3].

Of all the different forms of dementia, Alzheimer's disease (AD) is the most common [4]. AD is characterised by the presence of extracellular plaques composed of amyloid-beta (Aβ) protein, intraneuronal tau in hyperphosphorylated form and significant loss of synaptic connectivity and neuronal death leading to brain atrophy [5]. Aβ protein is derived from the cleavage of a transmembrane protein, amyloid precursor protein (APP), of which function has been related to a neuroprotective effect against traumatic brain injury [6,7]. Cleavage of APP by an α-secretase enzyme results in soluble APPα (sAPPα), the latter also being associated with neuroprotection [8]. Cleavage of APP by β-site APP-cleavage enzyme 1 (BACE1) generates soluble APPβ (sAPPβ) and the fragment C99 which is then subsequently cleaved by the γ-secretase enzyme complex to produce the insoluble Aβ42 peptide; the main constituent of extracellular plaques found in the brain of AD individuals [9,10]. Genome-wide association studies (GWAS) have identified more than thirty genomic loci that are associated with an increased risk of developing AD. These include *BIN1, CD2AP, CLU, CR1, EPHA1, PICALM, CD33, MS4A4E and TREM2* [11,12]. Interestingly a significant number of these loci are implicated in the inflammatory and neurophysiological changes that characterize the AD brain such as cholesterol and Aβ dysregulation and metabolism. For example, *PICALM* plays a central role in Aβ blood–brain barrier transcytosis and clearance [13] whereas *CD33*, mainly expressed in microglia cells, is thought to inhibit normal functions of immune cells [14] and impairs microglia-mediated Aβ clearance [15].

Mutations in genes that encode APP and γ-secretase have also been found to increase the susceptibility of developing AD, especially early on in life. Constituents of γ-secretase, presenilin-1 (PS1) and presenilin-2 (PS2), can enhance the cleavage of the multi-subunit γ-secretase complex thus facilitating the production of Aβ42 and the resulting plaques [16]. PS1 is typically associated with the familial form of AD and neurons expressing a mutated PS1 show a dysregulation of calcium ion signalling [17]. Presenilins are also involved in neuroprotection against oxidative stress [18] and thus PS mutants inhibit neuronal protection against toxic insults [19]. Other activities in which presenilins play an important role include inflammatory signalling [20], cellular differentiation [21], autophagy [22] and copper uptake [23].

The protein that has been mostly associated with late onset AD (LOAD) is apolipoprotein E (APOE), in particular, the allele ε4 of APOE (APOE4). APOE is a lipid carrier involved in the modulation of lipid transport and synaptic plasticity [24,25]. In humans, APOE is expressed in the brain in three isoforms that differ from each other in not more than two amino acids: APOE2, APOE3 and APOE4. The allele frequencies differ with ε3 being the most common followed by ε4 and ε2 [26]. However, epidemiological studies revealed that the risk for AD is up to three-fold higher in individuals with one APOE ε4 allele and about 12-fold higher in those with two APOE ε4 alleles [27]. The mechanism by which this occurs is still unclear but recent studies suggest that APOE4 has a direct effect on Aβ clearance [28]. Interestingly, carriers of two APOE ε2 alleles have a significant reduced risk of developing AD [29].

A number of genes have been implicated in other forms of dementia. Various investigations have found a significant association between APOE and vascular dementia (VaD) [30]. Other genes related to inflammatory response including those encoding tumour necrosis factor-α (TNF-α) [31], transforming growth factor beta 1 (TGF-β1) [32] and heat shock protein 70 [33] have also been associated with an increase in developing VaD. Between 20 and 50% of frontotemporal dementia (FTD) cases are familial [34] and characterised by mutations in microtubule-associated tau protein, granulin, valosin-containing protein, chromatin-modifying 2B, TARDNA binding protein 43 encoding gene, integral membrane protein 2B and tank-binding kinase 1 resulting in impairment in language, behaviour, memory and motor function [[35], [36], [37]]. Various studies have identified a number of genes that are implicated in familial Lewy body dementia (LBD), a neurodegenerative disease characterised by progressive impairment of cognitive function. These genes code for α-synuclein [38], leucine-rich kinase 2 [39], presenilins 1 and 2 [40,41], APP [42] and β-synuclein [43]. The fact that a number of these proteins are also associated with AD and Parkinson's disease indicates a clear genetic overlap between LBD and other neurodegenerative conditions [44].

In the last few years, a number of modifiable and non-modifiable risk factors have been identified for AD. Among the non-modifiable risk factors, increasing age is the most important. Females are at a higher risk of developing the disease compared to men as are individuals with trisomy of Chromosome 21 [45]. Clinical obesity, vascular disease, hypercholesterolemia, hypothyroidisms, diabetes, chronic alcohol abuse, smoking and repeated head trauma are among the modifiable risk factors [46]. Change in lifestyle habits including physical exercise and the consumption of a Mediterranean diet [47] can also protect against developing AD.

Over the last two decades, a number of pharmacotherapeutic agents have been licensed for the symptomatic management of the most common forms of dementia, particularly AD. All of these drugs target neurotransmitter dysfunction, with acetylcholinesterase inhibitors (AChEIs) being the first to be marketed for clinical use [48]. Studies showed that these drugs significantly improved cognition and activities of daily living in AD individuals [49]. Another drug is memantine, a glutamatergic system modifier that partially blocks the N-methyl-D-aspartate receptor channel leading to a reduction in calcium-induced cytotoxicity [50]. In randomised clinical trials, memantine demonstrated the ability to delay cognitive and functional decline without any significant side effects [51,52]. According to the latest guidelines issued by the British Association of Psychopharmacologists [53], AChEIs (donepezil, rivastigmine, and galantamine) are effective for cognition in mild to moderate AD and memantine for moderate to severe AD. Combination therapy (AChEIs and memantine) may also be beneficial in moderate to severe AD and can contribute to delaying admission to residential and nursing home care [54]. There is not enough evidence as yet that other drugs including statins [55], anti-inflammatory agents [56], hormone replacement therapies [57], vitamin E [58] and nutritional supplements such as gingko biloba [59] offer any benefits in the treatment and prevention of AD. AChEIs can also be used in the treatment of Lewy-body dementia [60] but not in frontotemporal dementia as these may cause increase behavioural challenges that usually occur with disease progression [61]. No effective treatment exists for vascular dementia.

## 2 Non-coding RNAs

The term non-coding RNA (ncRNA) refers to RNA sequences which do not have the ability to be translated into proteins and therefore mainly fall within the realm of the recently acknowledged ‘dark matter’ of the genome. This genomic dark matter encompasses a whole spectrum of differing ncRNA families such as microRNAs (miRNAs), long non-coding RNAs (lncRNAs), PIWI-interacting RNAs (piRNAs), transfer RNAs (tRNAs), small nuclear RNAs (snoRNAs) and circular RNAs (circRNAs), to name but a few [62]. However, the two most clinically valuable ncRNAs classes by far to be acknowledged would be the miRNA and lncRNA families. This is due to their immense capacity to invoke gene regulation in a very fine-tuned and effective manner across all cellular physiological pathways within humans and mammals. In addition, the level of scientific evidence to confirm the major gene regulatory roles of both ncRNA families within scientific literature in the past two decades is astoundingly solid. Consequently, due to the widespread influences of miRNAs and lncRNAs across all disease pathways, it is of critical importance for researchers in the field of dementia to focus their attention on possible ncRNA-induced pathogeneses, with the ultimate goal of identifying novel diagnostic procedures and drug targets, together with the development of novel therapies to control such a devastating mental condition in the patient population.

## 3 Influence of miRNAs in dementia

The description of a typical mature miRNA is that of a single 22 nucleotide RNA sequence, with this active strand having near-complementary to the 3′ untranslated region (3’ UTR) of the designated target messenger RNA (mRNA) [63]. Once attached, the miRNA acts as a physical obstruction for ribosomal action and therefore inhibits the process of translation of the protein/s coded by that specific mRNA transcript [63]. Due to their mode of action, miRNAs are typically described to act as post-transcriptional gene regulators [63]. There exist approximately 2600 members in the miRNA family and dys-regulated expression of even just a single miRNA can lead to the development or aggravation of a plethora of clinical conditions, particularly in cancer [64]. Consequently, the identification and validation of specific miRNAs having a direct or indirect involvement in the pathogenesis and/or disease progression of all forms of dementia has also been well recognised in the scientific literature. The following sections briefly describe landmark studies that undoubtedly demonstrate the effects of miRNA dysregulations on the multiple forms of dementia.

### 3.1 AD

The first seminal research findings that elucidate correlation of miRNA dysregulation with AD date back to 2008 and were carried out by Cogswell and colleagues [65]. This study identified specific dys-regulations in miRNA expression profiles within AD-afflicted cerebral tissues and cerebro-spinal fluid (CSF) [65]. Another study carried out in the same year, carried out by Hebert and colleagues, led to the discovery of a loss of the miR29a/b-1 cluster within sporadic AD cases [66]. In addition, this study also demonstrated the direct influence of miR-29a, -29b-1 and -9 in regulating in vitro expression of BACE1 and members of this miRNA cluster were inversely correlated to APP expression [66]. A similar study also demonstrated that down-regulated miR-29a expression allowed for the exacerbated expression of neurone navigator 3 (NAV3), which is an axon guiding regulator and also a direct target gene of miR-29a, with NAV3 typically being up-regulated in AD cerebral tissue samples [67]. Furthermore, evidence of miRNA involvement in APP regulation were highlighted by Long and Lahiri in 2011, whereby this study elucidated the regulatory effects of miR-101 on APP expression within human cell cultures such as HeLa and SK-N-SH [68].

Further evidence for the influence of miRNAs in BACE1 expression was elucidated by the investigations carried out by Boissonneault and colleagues in 2009 [69]. This study concluded that miR-298 and miR-328 effectively regulate BACE1 expression within murine models of AD [69]. A more recent study also identified miR-124 as capable of targeting BACE1 expression and with the added capacity of influencing the level of autophagy within APP/PS1 transgenic murine models [70]. The effects of miR-124 on BACE1 were also confirmed by a later study [71]. Additional evidence of miRNA-induced BACE1 regulation was also provided through the study carried out by Gong and colleagues, which investigated the effects of miR-15b on BACE1 expression [72]. The results of this study included the revelation that miR-15b was down-regulated within brain samples of sporadic AD patients, with an inversely proportional increase in BACE1 expression, suggesting that BACE1 is also targeted for regulation by miR-15b [72].

In 2010, the in vitro and murine study performed by Wang and colleagues elegantly highlighted the regulatory role of miR-107 expression on granulin (GRN/progranulin) – a protein that is widely recognised to induce frontotemporal dementia and AD [73,74]. Another study identified miR-27a-3p as a potential AD biomarker, as it was found to be down-regulated within 35 AD patient CSF samples following RT-qPCR analysis [75]. More recent evidence was highlighted by the study carried out by Higaki and colleagues [76]. These investigations revealed that members of the miR-200 family, namely miR-200b and miR-200c, were up-regulated following the increased presence of Aβ in murine primary neurons [76]. In addition, the cerebral infusion administration of both miRNAs into murines having oligomeric amyloid beta-induced memory constraints was found to allow memory recovery and cognitive abilities in the murines [76]. These results confirm the neuroprotective and defence mechanism abilities of miR 200-b and miR 200-c in reverting the AD condition [76]. Other miRNAs found to have a neuroprotective effect against amyloid-beta presence in AD include miR-431 [77]. This study, conducted by Ross and colleagues, demonstrated that miR-431 regulated Kremen1 expression, the latter being a transmembrane receptor for Dickkopf-1 (DKK1) – a major AD pathogenesis player due to its effect in exacerbating synaptic breakdown through Aβ activity [77].

Consequently, these studies described above all recognise that specific miRNA expression profiles impose a regulatory role on AD-inducing genes, with a down-regulation of such miRNA network allowing exacerbated expression of such genes and their corresponding AD-inducing proteins. Conversely, apart from experimental validation of AD-suppressing miRNA activities, other studies have also highlighted the existence of detrimental, AD-inducing miRNAs – as described below and in Table 1.

**Table 1:** Compendium of the most relevant miRNAs identified to influence dementia, either as pathology-beneficial or detrimental. AD – Alzheimer's Disease, VaD – vascular dementia, LBD – lewy body dementia, FTD – frontotemporal dementia.

miRNA/s involved	Dementia form	Functional role of miRNA/s (when up-regulated)	Affected pathways and/or gene/s	Reference/s
miR-29a/b-1 cluster	AD	Beneficial	BACE1	[66]
miR-29a	AD	Beneficial	NAV3	[67]
miR-124	AD	Beneficial	BACE1	[70,71]
miR-200b/c	AD	Beneficial	Neuroprotective	[76]
miR-125b	AD	Detrimental	P35/25 pathway	[81]
miR-322 cluster	AD	Detrimental	BDNF	[82]
miR-29b	FTD	Beneficial	Progranulin	[90]
miR-34a	VaD	Detrimental	Bcl-2	[86]

One of the first studies to elucidate AD-inducing miRNA activity was the investigation carried out by Li and colleagues in 2011 [78]. This study utilised multiple human primary brain/retinal cell lines together with five transgenic murine models of AD to analyse miR-146a expression across all three AD stages (early, moderate and late) [78]. The results of this investigation concluded that exacerbation of miR-146a leads to AD development, with increased miR-146a expression levels being correlated to increased plaque density within all five transgenic murine AD models [78]. In a similar study, Wang and colleagues identified miR-106b to be up-regulated in a double transgenic murine AD model, with miR-106b directly regulating TGF-β type II receptor and ultimately thwarting TGF- β signalling [79]. Furthermore, the study conducted by Zovooilis and colleagues described miR-34c to play a crucial role in debilitating learning ability and other cognitive faculties within the hippocampus of AD murine models and AD patients due to its up-regulated levels in such models [80].

More recent studies include the investigations carried out by Ma and colleagues, which focused on the effects of miR-125b in AD [81]. This study discovered a consistent up-regulated state in AD patients and that miR-125b up-regulation led to neuronal apoptosis and tau phosphorylation status through exacerbation of the cyclin-dependent kinase 5 (CDK5) and p35/25 pathways [81]. Incidentally, other miRNAs that lead to tau phosphorylation include the miR-322 cluster [82]. This study elucidated an up-regulation (within murine models) of miR-322 and inversely proportional down-regulation of brain-derived neurotrophic factor (BDNF), the latter being a typical occurrence in AD patients [82]. In essence, the study revealed that miR-322 induced tau phosphorylation through regulation of the BDNF-TrkB receptor complex activation by down-regulation of BDNF [82]. Exacerbated expression of miR-200a-3p was also found to regulate SIRT1, ultimately leading to an increase in Aβ-induced neuronal apoptotic status within the hippocampus of APPswe/PE delta E9 murine models for AD [83]. Further results presented by Hadar and colleagues revealed that SIRT1 expression is also regulated by miR-132 and miR-212 on analysis of post-mortem olfactory bulb and hippocampus samples from 14 AD patients [84].

### 3.2 VaD/LBD/FTD

The current evidence available in the scientific literature for the influences of miRNAs on VaD is presently much more scarce, as most focus by global research groups is directed towards AD research.

Involvement of miRNAs in VaD includes the recent study by Chen and colleagues on chronic brain hypoperfusion (CBH) – a hallmark finding in vascular dementia that leads to neuronal loss and a reduction in dendritic interconnectivity in rat hippocampi/cortices [85]. This study identified miR-195 to exacerbate the N-terminal B-amyloid precursor protein/death receptor-6/caspase signalling pathway, ultimately leading to aggravation of CBH-induced dendritic breakdown [85]. Another landmark study conducted by Liao and colleagues recognised the detrimental influences provoked by miR-34a up-regulation on Bcl-2, with inhibition of this miRNA allowing for vascular endothelial cell protection from VaD, brain oedema and also arteriosclerosis [86].

The influences of miRNA dysregulations in LBD have not been delved into greater detail as yet, though the landmark study by Pietrzak and colleagues in 2016 has led to the extrapolation of 22 miRNA binding sites following the identification of a 490-gene expression profile in post-mortem cerebral tissues of eight LBD dementia patients [87]. A similar study carried out by Nelson and colleagues in the same year focused on performing RT-qPCR-based miRNA microarrays on anterior cingulate and primary motor cortical tissue from post-mortem patients with confirmed dementia with Lewy bodies [88]. Further validation analyses of a subset of these miRNA microarray results confirmed a dysregulated expression profile in the LBD patient group, consisting of miR-7, miR-153, miR-133b, miR-137 and miR-34a [88].

Interestingly, evidence of miRNA Influences in FTD is more prevalent than for LBD or VaD. The first documented study dates back to 2008 and was conducted by Rademakers and colleagues [89]. This study elucidated the negative regulatory function of miR-659 on progranulin expression, which ultimately leads to TAR DNA-binding protein 43-/ubiquitin-positive FTD [89]. Additional progranulin negative regulation is through exacerbated miR-29b expression [90], miR-107 [74] and the miR-132/212 cluster [91]. Furthermore, the study carried out by Zhang and colleagues in 2013 identified the downregulation of miR-9 in FTD patients, suggesting that miR-9 expression has a neuroprotective effect on this form of dementia [92]. Multiple studies have also identified and validated miR-124 to induce social behaviour dysfunctions in FTD [93,94]. In addition, the recently published research by Jawaid and colleagues revealed that dysregulation of three miRNAs (miR-183, miR-96 and miR-182) concomitantly were correlated to FTD in vivo [95].

## 4 Influence of lncRNAs in dementia

Individual lncRNA can be described as single RNA sequences of at least 200 nucleotides in length [64]. The entire lncRNA family is currently composed of over 60,000 members, with individual lncRNA having the capacity to be either negative or positive regulators for their corresponding target genes [64]. Furthermore, the gene modulating functions of lncRNAs can occur either on distant chromosomal loci (trans-acting) or on the actual chromosomal locus housing the specific lncRNA (cis-acting) [64]. A summary of the most relevant lncRNAs in dementia are listed in Table 2 below.

**Table 2:** Compendium of the most relevant lncRNAs identified to influence dementia, either as pathology-beneficial or detrimental. AD – Alzheimer's Disease, VaD – vascular dementia, LBD – lewy body dementia, FTD – frontotemporal dementia.

lncRNA/s involved	Dementia form	Functional role of lncRNA/s (when up-regulated)	Affected pathway and/or gene/s	Reference/s
BACE1AS	AD	Detrimental	BACE1	[96,97]
BC200 RNA	AD	Detrimental	Synaptic RNA delivery	[99]
MALAT1	AD	Detrimental	Neuronal apoptosis	[103]
NEAT1_2/MALAT1	FTD	Detrimental	TDP-43	[107]

### 4.1 AD

The seminal study by Kang and colleagues elucidated the effect of the lncRNA BACE1 antisense (BACE1AS) in exacerbating BACE1 expression [96]. In addition, the study conducted by Liu and colleagues also demonstrated that silencing of BACE1AS leads to an inability by BACE1 to cleave APP within neuroblastoma tumour-AD cell line models, therefore thwarting the aggravated development of senile plaques [97]. The research carried out by Magistri and colleagues delved into the possible correlation between dysregulated lncRNA expression profiles and LOAD [98]. The results of this study revealed that 14 differing lncRNAs are typically up-regulated and enriched in LOAD-afflicted neuronal cells [98]. The lncRNA BC200 RNA was also identified as a contributor to AD through its up-regulated expression and consequent lack of synaptic RNA delivery [99]. In another investigation, the lncRNA BC1 was revealed to contribute to the spatial learning and memory deficit facets of AD progression, through its ability to induce APP mRNA translation within the brains of AD murines [100]. Another study, conducted by Gu and colleagues, recognised the lncRNA early B cell factor 3 antisense (EBF3-AS) to be highly up-regulated within the hippocampus of APP/PS1 murine models and the same lncRNA had the effect of exacerbating neuronal apoptotic mechanisms [101]. Incidentally, BDNF-AS and MALAT1 regulate the same apoptotic pathways as EBF3-AS in order to induce neuronal toxicity in PC12 cells [102,103]. The lncRNA SOX21-AS1 was also recognised to induce neuronal oxidative stress injuries in murine AD models, through influences on the Wnt signalling pathway once SOX21-AS1 is up-regulated [104].

However, neuroprotective lncRNAs in AD have also been reported within scientific literature. The study by Jiang and colleagues identified MIAT having the capacity to regulate vascular dysfunctions including vascular permeability, through the MIAT/miR-150-5p/VEGF signalling pathway [105]. MIAT knockdown in this study led to neurone degeneration, cerebral microvasculature dysfunction and behavioural issues in AD murine models [105]. In another recent study, human urothelial carcinoma associated 1 (UCA1) was found to have deep-seated influences in neuronal stem cell differentiation, mainly through the regulation of the miR-1/Hes1 pathway, with UCA1 knockdown leading to a reduction in neuronal stem cell proliferation [106].

### 4.2 VaD/LBD/FTD

Unexpectedly, the level of research on lncRNA involvement in other forms of dementia is still in a vacuum, with only one study that identified NEAT1_2 and MALAT1 to bind with higher degrees to TDP-43 within cases of FTD [107].

## 5 Conclusions and perspectives

Undoubtedly, the widespread influences of non-coding RNAs have found to be ramified in all aspects of human disease, including neurodegenerative disorders that lead to syndromes such as dementia. The current body of research veers towards the discovery of novel roles for non-coding RNA family members in the pathogenesis and clinical development of AD, since it is the most prevalent form of dementia within the global population. Nevertheless, revelations into the roles of non-coding RNA – in particular lncRNAs - within all other forms of dementia are still emerging within the scientific literature. The clinical value for the identification and validation of such non-coding RNAs is the revelation of novel drug targets for future translational medicine therapeutic approaches. However, since both miRNAs and lncRNAs are also present within circulating and isolated body fluids, these can also act as diagnostic and disease-stratifying biomarkers for dementia through basic liquid biopsies such as blood sampling [[108], [109], [110], [111], [112], [113]].

The authors truly believe that such novel approaches to clinical theranostics for dementia, similar neurodegenerative disorders and other human disease conditions are expected to emerge within the next decade and become one of the mainstay pillars of future medical care for the global patient population.

## Conflicts of interest

The authors declare that there are no competing interests.
